# Nationwide Trends in Hospital-Acquired Pressure Ulcers, 2018–2024

**DOI:** 10.3390/healthcare14111492

**Published:** 2026-05-27

**Authors:** Emanuele Sebastiani, Danilo Catania, Stefano Domenico Cicala, Massimo Maurici, Michele Tancredi Loiudice, Giovanni Baglio

**Affiliations:** 1 Department of Biomedicine and Prevention, University of Rome Tor Vergata, 00133 Rome, Italy; 2 Research and International Relations Unit, Italian National Agency for Regional Healthcare Services, 00187 Rome, Italy; 3 Simple Operative Unit Statistics and Health Information Flows, Italian National Agency for Regional Healthcare Services, 00187 Rome, Italy

**Keywords:** pressure ulcers, patient safety indicators, nursing-sensitive outcomes, hospital discharge records, quality of care

## Abstract

**Aim:** To estimate national incidence, temporal trends, and regional variability of hospital-acquired pressure ulcers in Italy from 2018 to 2024 using age-adjusted models and regional estimates. **Design:** Retrospective nationwide observational study using hospital administrative data. **Methods:** All Italian Hospital Discharge Records (SDOs) for adults aged ≥ 18 years with hospital stays ≥ 5 days between 2018 and 2024 were analysed. Records with pre-existing or principal diagnoses of pressure ulcer and excluded MDC/DRG categories were omitted according to adapted AHRQ PSI 03 specifications. The final dataset represented eligible hospitalizations considered at risk for hospital-acquired pressure ulcers. Crude and age-adjusted rates per 10,000 eligible discharges were estimated using logistic regression models. **Results:** Eligible discharges declined from approximately 1.7 million in 2018 to 1.4 million in 2020, increasing to 1.5 million in 2024. Within this population, coded hospital-acquired pressure ulcer events decreased from 3657 to 1888, then increased to 2728. Age-adjusted national rates ranged from 13.5 to 21.3 per 10,000 eligible discharges, showing temporal fluctuations during the study period, including a reduction during 2020–2021 followed by a gradual return toward pre-pandemic levels. Substantial regional variability was observed, with lower median annual adjusted rates in regions such as Friuli Venezia Giulia and Toscana and higher values in Lazio and Abruzzo. **Conclusions:** This nationwide analysis provides an initial descriptive overview of temporal and regional variability in coded hospital-acquired pressure ulcer events identified through an adapted PSI 03-based administrative indicator in Italy. The findings may contribute to future methodological discussion and exploratory development of nursing-sensitive indicators using national administrative healthcare databases. **Implications for the profession and/or patient care:** The integration of nursing-sensitive administrative indicators into national quality monitoring systems may represent an initial methodological area for future benchmarking activities, indicator validation processes, and descriptive evaluation of preventive care practices using national healthcare administrative databases. **Impact (addressing):** Problem: limited national evidence on hospital-acquired pressure ulcers and interregional variability in Italy. Main findings: temporal fluctuations in age-adjusted rates and persistent regional heterogeneity in coded pressure ulcer events. Impact: administrative data may represent a preliminary and exploratory source for the study of nursing-sensitive outcomes and patient safety indicators at national level.

## 1. Introduction

Healthcare systems worldwide are facing increasing challenges due to population ageing, rising demand for care, escalating costs, and the constant need to ensure patient safety and quality outcomes. Improving care quality requires systematic performance measurement and outcome monitoring across care pathways.

In Italy, the National Outcomes Programme (Programma Nazionale Esiti, PNE) provides a key national framework for assessing the quality, equity, and efficiency of the Servizio Sanitario Nazionale and for ensuring transparency to citizens [1]. Within this framework, nursing-sensitive outcomes (NSOs) are defined as measurable changes in patient health potentially influenced by nursing care [2,3].

NSOs are increasingly recognized as important indicators for the descriptive evaluation of healthcare quality and patient safety.

Among NSOs, pressure ulcers are one of the most widely recognised indicators potentially associated with patient safety and nursing care quality. They are areas of localized skin and tissue damage, usually over bony prominences, resulting from sustained pressure or shear forces [4]. Pressure ulcers are associated with significant clinical and economic consequences, including increased infection risk, pain, functional decline, longer hospital stays, and elevated mortality rates, as well as higher healthcare costs [5,6].

Their occurrence also has important organizational implications, contributing to increased healthcare resource utilization and burden on healthcare systems.

However, pressure ulcer development is influenced by multiple clinical, organizational, and patient-related factors, including immobility, nutritional status, frailty, comorbidities, and care complexity [7,8].

International evidence confirms their epidemiological relevance: a meta-analysis in intensive care settings estimated an incidence of up to 10% [9], while a global systematic review reported an overall hospital prevalence of 12.8% and an incidence of 5.4 per 10,000 patient-days [10]. These data underscore the importance of standardised monitoring and preventive strategies at the national level.

The Agency for Healthcare Research and Quality (AHRQ) Patient Safety Indicators (PSIs) are internationally recognised measures developed to identify potentially preventable adverse events using administrative hospital data. Among them, PSI 03 (Pressure Ulcer Rate) [11] has been widely adopted for quality monitoring and benchmarking activities due to its standardisation and applicability to large administrative databases. Nevertheless, the implementation and interpretation of PSI-based indicators remain methodologically challenging, particularly in healthcare systems where administrative databases were not originally designed as nursing-sensitive clinical information systems.

In the Italian context, the present study also aims to explore the applicability of an adapted AHRQ PSI 03-based administrative indicator as a preliminary and exploratory nursing-sensitive outcome indicator within the framework of the National Outcomes Programme.

Nevertheless, the use of administrative healthcare data presents important methodological limitations. Hospital Discharge Records (Schede di Dimissione Ospedaliera, SDOs) [12] do not provide detailed clinical chart information and only capture diagnoses recorded within discharge coding systems. Consequently, pressure ulcer identification depends on the accuracy and completeness of coding practices. Furthermore, several clinically relevant determinants commonly associated with pressure ulcer development—including functional status, mobility impairment, nutritional condition, ulcer staging, and detailed nursing assessment variables—are not available within the Italian SDO administrative database.

However, the interpretation of administrative healthcare data should also consider the specific characteristics of the Italian National Health Service (SSN) reimbursement system. In Italy, hospital funding is largely based on Diagnosis-Related Groups (DRGs), where secondary diagnoses such as Hospital-Acquired Pressure Ulcers (HAPUs) may contribute to the classification of complications or comorbidities and potentially influence the DRG weight assigned to hospitalization episodes. Consequently, hospital administrative departments and clinical coders may have an operational incentive to accurately document these conditions within hospital discharge records (SDOs). Although this does not eliminate the intrinsic limitations of administrative databases or regional heterogeneity in coding practices, it may partially reduce the likelihood of systematic under-reporting and support the validity of longitudinal analyses based on national SDO data.

Moreover, the current Italian ICD-9-CM classification system does not consistently support pressure ulcer staging codes included in later ICD-9-CM revisions, thereby limiting the clinical granularity of the analysis. Therefore, this study should be considered a preliminary population-based exploratory approach that may support future methodological validation studies comparing administrative indicators with detailed clinical data in specific healthcare settings. However, in Italy, an internationally recognised administrative indicator potentially applicable to nursing-sensitive outcomes has not yet been applied to national administrative data. To date, no nationwide study has systematically applied an adapted AHRQ PSI 03-based indicator to the complete Italian Hospital Discharge Records database for the evaluation of hospital-acquired pressure ulcers as a nursing-sensitive outcome.

This study addresses that gap by analysing all Hospital Discharge Records (Schede di Dimissione Ospedaliera, SDOs) from 2018 to 2024, using an adapted AHRQ PSI 03-based administrative indicator to estimate national incidence, temporal trends, and regional variability.

The findings may provide preliminary epidemiological and methodological evidence to support future research on nursing-sensitive outcome indicators and their potential exploratory applicability within the Italian healthcare performance evaluation system.

## 2. Materials and Methods

### 2.1. Design

This study adopted a retrospective, nationwide, observational design using administrative hospital data. The analysis followed the Strengthening the Reporting of Observational Studies in Epidemiology (STROBE) statement for cross-sectional studies [13].

### 2.2. Data Source and Study Population

Data were obtained from all Schede di Dimissione Ospedaliera (SDO; Hospital Discharge Records) collected by the Italian Ministry of Health between 2018 and 2024 [12]. Each record includes anonymised patient-level information on demographics, principal and secondary diagnoses, performed procedures, discharge status, and length of stay. Hospitalizations were selected according to inclusion and exclusion criteria defined by the Agency for Healthcare Research and Quality (AHRQ) for Patient Safety Indicator (PSI) 03: Pressure Ulcer Rate [11].

A detailed reproducible flow of the cohort selection process, including all ICD 9-CM codes, DRG/MDC exclusions, transfer exclusions, POA management, and the number of hospitalizations excluded at each step, is reported in Supplementary Table S1.

Eligible hospitalizations included ordinary inpatient admissions among adult patients aged ≥ 18 years with a length of stay of at least 5 days, in accordance with AHRQ technical specifications. Exclusion criteria were:

(a) principal diagnosis of pressure ulcer on admission;
(b) age under 18 years;
(c) missing or implausible information regarding sex, diagnosis codes, or length of stay,
(d) pressure ulcers coded as present on admission (POA);
(e) admissions related to MDC 09 (diseases and disorders of the skin, subcutaneous tissue and breast) and MDC 14 (pregnancy, childbirth and the puerperium);
(f) selected non-eligible DRGs according to AHRQ PSI 03 specifications;
(g) admissions from long-term care facilities or transfers from acute care hospitals;
(h) selected neurological and paralytic conditions associated with high baseline pressure ulcer risk;
(i) selected operating room procedures, including debridement and skin graft procedures performed as principal procedures or during the same operative episode.

Transfer exclusions were implemented using admission source variables available in the SDO database, consistent with AHRQ PSI 03 specifications for excluding transfers from acute care hospitals and long-term care facilities.

Specific admission source codes used for transfer and long-term care exclusions included SDO provenance codes 05, 06, 07, 08, and 10, consistent with the adapted AHRQ PSI 03 algorithm implemented in SAS 9.4.

The POA variable available in the SDO database was used to exclude pressure ulcers already documented at hospital admission from the numerator definition. The POA variable was analysed for each secondary diagnosis field in order to identify pressure ulcers documented as present at admission and to exclude them from the hospital-acquired event definition.

Pressure ulcers identified through ICD-9-CM code 707.0x in secondary diagnosis positions were excluded from the numerator when the corresponding POA flag indicated presence at admission.

A preliminary evaluation of POA coding completeness and consistency was also performed at the regional level in order to assess the comparability of reporting practices and reduce the potential risk of misclassification associated with heterogeneous administrative coding quality.

Because the SDO database is fully anonymised, repeated admissions for the same patient could not be longitudinally linked and were therefore analysed as independent hospitalization episodes.

After applying these criteria, approximately 1.4–1.7 million eligible hospitalizations per year were included in the analytic denominator.

### 2.3. Indicator Definition

The indicator was based on the Agency for Healthcare Research and Quality (AHRQ) Patient Safety Indicator 03 (Pressure Ulcer Rate) technical specifications and adapted to the structure of the Italian SDO administrative database. Hospital-acquired pressure ulcers were identified using ICD-9-CM secondary diagnosis codes 707.0x, including codes 70700–70709 for decubitus ulcers, in accordance with AHRQ technical specifications [11]. ICD-9-CM staging codes 707.2x were not consistently available within the Italian ICD-9-cm 2007 coding system adopted in the national SDO database and were therefore not included in the case definition.

Consequently, the indicator should be considered an adapted PSI 03-based administrative indicator rather than a direct replication of the original AHRQ PSI 03 specification. The adapted indicator was designed to maintain maximum comparability with the original AHRQ PSI 03 framework while accounting for the structural limitations of the Italian ICD-9-CM 2007 coding system.

The numerator comprised discharges with at least one qualifying secondary diagnosis of pressure ulcer, while the denominator included all eligible discharges after exclusions.

Pressure ulcer cases were identified only when recorded in secondary diagnosis fields, excluding primary diagnosis positions, in order to approximate hospital-acquired events.

Crude and risk-adjusted rates were expressed as the number of events per 10,000 hospital discharges. Risk adjustment and eligibility criteria were implemented through SAS 9.4 [14] algorithms reproducing the AHRQ PSI 03 technical specifications adapted to the Italian SDO administrative database structure.

The full SAS coding logic used to implement the adapted PSI 03 algorithm included sequential exclusion procedures for non-eligible DRGs, MDC categories, transfers, neurological conditions, debridement/skin graft procedures, operating room procedures, and POA-based filtering.

It should be noted that Italy still applies the 2007 version of ICD-9-CM, which does not include ulcer staging introduced in later revisions, thereby limiting comparability with international studies using updated classifications.

### 2.4. Risk Adjustment Model

Risk adjustment was performed using a logistic regression model implemented in SAS 9.4 [14], within a generalized linear model framework with binomial distribution and logit link function.

Age was included as the only covariate, as advanced age has consistently been recognized as a robust and epidemiologically relevant risk factor associated with pressure ulcer development in hospitalized and population-based studies [7,8]. Moreover, age represents one of the most consistently available and methodologically reproducible variables within administrative healthcare databases.

The choice of a parsimonious adjustment model was also influenced by the characteristics of the Italian SDO database, which was not originally designed as a clinical or nursing-sensitive information system.

In particular, several clinically relevant determinants commonly associated with pressure ulcer development—including functional status, mobility impairment, nutritional status, ulcer staging, and standardized nursing assessment variables—are not available within the SDO administrative database.

Additional variables, including length of stay, clinical discipline, and admission source, were explored descriptively in dedicated analyses rather than included directly in the adjustment model, as these factors may also represent intermediate organizational or care-related variables rather than pure baseline confounders.

Their inclusion could therefore have introduced overadjustment or attenuated clinically meaningful differences.

Accordingly, the present model should be interpreted primarily as a descriptive standardization approach rather than a fully comprehensive causal risk-adjustment model. Substantial residual confounding likely remains, particularly with respect to unmeasured clinical severity, frailty, comorbidity burden, functional impairment, and organizational characteristics not captured within the administrative database.

### 2.5. Regional and Descriptive Analyses

Risk-adjusted rates were computed separately for each Italian region. Interregional variability was assessed using descriptive statistics and visualised through boxplots. The boxplots represented the distribution of annual regional risk-adjusted rates observed between 2018 and 2024, with each data point corresponding to a region-year observation.

Additional analyses examined length of stay (LOS) by year, age group, and clinical discipline, according to the SDO classification system, to explore the organisational and patient-level characteristics associated with pressure ulcers. These variables were analysed descriptively and were not included in the primary risk-adjustment model.

An exploratory non-linear analysis was also performed to evaluate possible temporal inflections during the COVID-19 period using PROC NLIN in SAS with a spline-based interpolation approach. This analysis was interpreted exploratively and was not used for formal causal inference or joinpoint identification.

### 2.6. Ethical Considerations

This study was conducted using anonymised administrative data publicly released by the Italian Ministry of Health. No individual identifiers were available; therefore, ethical approval and informed consent were not required under national and European regulations [15]. The main analytical components, data source, indicator definition, cohort selection process, and statistical specifications adopted in the study are summarised in Table 1.

## 3. Results

### 3.1. Risk-Adjusted Rates and Temporal Trends

The analysis included all national Hospital Discharge Records (SDOs) from 2018 to 2024. After applying the inclusion and exclusion criteria defined by the adapted AHRQ PSI 03-based methodology implemented for the Italian SDO administrative database, the denominator comprised approximately 1.4–1.7 million eligible hospitalizations per year (see Table 2).

Risk-adjusted rates were computed using a logistic regression model (SAS 9.4) with a logit link and binomial distribution, adjusted for age (continuous).

Rates were standardized by 10,000 eligible discharges and recalibrated using the national observed-to-expected ratio (k coefficient). Ninety-five per cent confidence intervals (CIs) were estimated using Wald limits. At the national level, both crude and risk-adjusted rates of hospital-acquired pressure ulcers showed marked temporal variation.

The number of reported events declined from 3657 in 2018 to 1888 in 2020, followed by a gradual increase to 2728 in 2024. Crude rates decreased from 21.1 per 10,000 discharges in 2018 to 13.3 in 2020, remained relatively stable through 2021–2023, and rose again to 18.2 in 2024. Adjusted rates mirrored this pattern, ranging from 13.5 in 2020 to 21.3 in 2018, reaching 17.9 in 2024.

**Figure 1:**
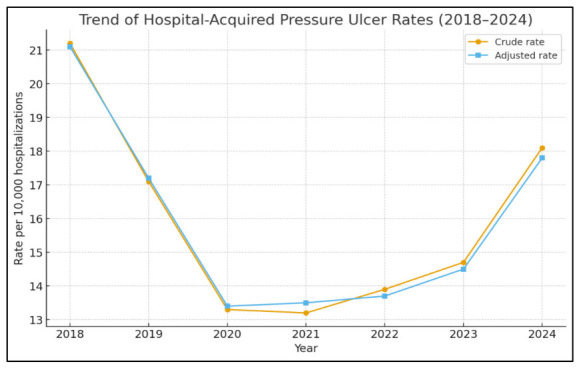
National trend of crude and risk-adjusted rates of hospital-acquired pressure ulcers based on the adapted AHRQ PSI 03 administrative indicator, Italy, 2018–2024. Source: Authors’ elaboration based on the Italian National Hospital Discharge Records (SDO), 2018–2024 [12].

Overall, the adapted PSI 03-based administrative indicator showed a temporal decrease during the PSI-based period, potentially consistent with altered hospitalization patterns and service reorganization, and a subsequent return toward pre-pandemic levels (Figure 1). However, due to the administrative nature of the dataset, these temporal variations cannot be directly attributed to changes in prevention quality, organizational performance, or clinical practice patterns alone.

### 3.2. Regional Variability

**Figure 2:**
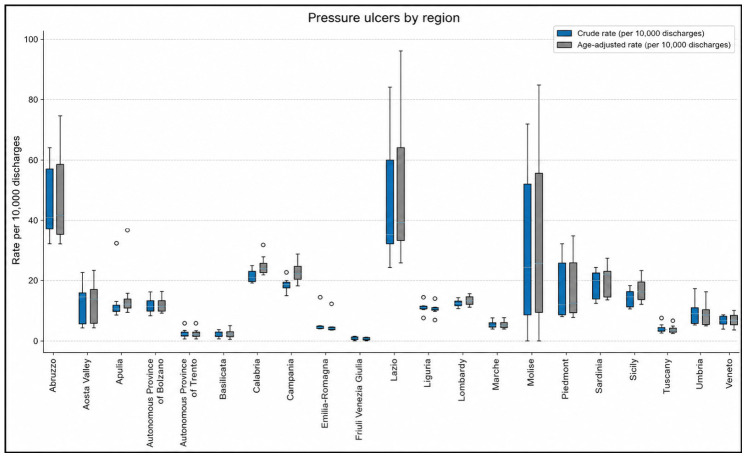
The boxplots summarize the distribution of annual regional crude and age-adjusted rates observed during the 2018–2024 period using the adapted AHRQ PSI 03-based administrative indicator. Each region contributed up to seven annual observations to the analysis. source: authors’ elaboration based on the Italian national hospital discharge records (SDO), Italy, 2018–2024 [12].

Risk-adjusted rates showed substantial heterogeneity across Italian regions. The median of the annual regional age-adjusted rates was approximately 14.0 per 10,000 eligible discharges, but wide territorial variability was observed (see Table 3; Figure 2).

The boxplots presented in Figure 2 were constructed using the annual regional crude and age-adjusted PSI 03 rates observed between 2018 and 2024. Each region, therefore, contributed up to seven annual observations to the distribution displayed in the boxplot, representing the temporal variability of rates within each region during the study period.

Table 3 summarizes cumulative crude rates and the distribution of annual regional risk-adjusted rates observed during the study period. Regions such as Friuli Venezia Giulia, Tuscany, Marche, Emilia-Romagna, and Veneto consistently reported the lowest median annual adjusted rates, whereas Lazio, Abruzzo, Molise, Calabria, and Campania showed substantially higher values and wider annual variability.

Lazio presented the highest median annual adjusted rate, with marked fluctuations across the study period (range: 26.3–94.0 per 10,000 discharges). Conversely, Friuli Venezia Giulia showed the lowest and most stable rates over time (range: 0.2–1.5 per 10,000 discharges). Overall, the observed variability between regions was substantial, with annual adjusted rates ranging from 0.2 to 94.0 per 10,000 eligible discharges across the study period. However, these regional differences should be interpreted cautiously, as administrative data may also reflect variability in coding practices, patient complexity, healthcare organization, and regional reporting systems not fully captured by the adjustment model.

### 3.3. Descriptive Analysis of Hospital Stays

Nationally, 17,339 hospitalizations with pressure ulcers were identified between 2018 and 2024. The mean length of stay (LOS) was 21.7 days (95% CI: 21.4–22.1), showing temporal and demographic variation (see Table 4). The LOS remained stable in 2018–2019 (20.97 and 20.13 days, respectively), increased during the COVID-19 pandemic (23.83 in 2020; 24.24 in 2021), and subsequently decreased to 20.81 days in 2024, potentially reflecting the progressive normalization of hospital operations after the pandemic period.

### 3.4. Clinical and Disciplinary Context

Hospitalizations were concentrated on a limited number of clinical specialties (see Table 5). General Medicine accounted for 9722 cases (56%), followed by Geriatrics (2752; 16%) and Physical Medicine and Rehabilitation (1307; 8%), representing nearly 80% of the national total. The longest mean stays were recorded in Neurorehabilitation (104.8 days), Long-Term Care (46.5 days), and Psychiatry (42.0 days), potentially reflecting greater clinical complexity and chronic care needs among these patients.

### 3.5. Synthesis of Findings

Overall, the analysis suggested temporal and regional variability in the coded pressure ulcer events identified through the adapted PSI 03 administrative indicator. The PS indicator suggested a partial reduction in interregional variability over time.

Variations by age group and discipline reflect the structural heterogeneity of Italian hospitals and may support the importance of integrated multidisciplinary care for frail and long-stay patients.

## 4. Discussion

### 4.1. Main Findings and Interpretation

This nationwide analysis over the 2018–2024 period suggests that hospital-acquired pressure ulcers identified through the adapted PSI 03 administrative indicator remain a relevant nursing-sensitive outcome within the Italian administrative healthcare context. The observed patterns may reflect a combination of patient clinical complexity, hospitalization characteristics, organizational factors, and administrative coding dynamics.

National PSI risk-adjusted rates demonstrated temporal fluctuations, declining during 2020–2021—coinciding with the COVID-19 pandemic—and gradually returning toward pre-pandemic levels thereafter. Although no statistically significant joinpoints were identified, the overall pattern suggests stabilization of rates in recent years.

These findings may reflect the sensitivity of pressure ulcer rates to healthcare system reorganization and administrative reporting dynamics during the study period. However, given the administrative nature of the dataset, the observed temporal variations should not be interpreted as direct evidence of changes in prevention quality, healthcare performance, or patient safety outcomes.

The prior literature has suggested that nursing-sensitive indicators may vary according to healthcare system strain, workforce characteristics, and resource availability [2,5,16], although these relationships could not be directly evaluated within the present study design.

### 4.2. Geographical and Temporal Variability

Substantial interregional variability was observed across Italian regions, with some regions consistently reporting lower and more homogeneous rates than others. Northern regions generally showed lower rates, whereas several central and southern regions exhibited higher and more variable values, with Lazio consistently reporting the highest median annual adjusted rates during the study period.

These differences may reflect a combination of organizational characteristics, patient case-mix complexity, administrative coding practices, reporting variability, and regional healthcare system structures not fully captured within the adjustment model. Accordingly, the observed variability should not be interpreted as direct evidence of differences in healthcare quality or preventive effectiveness. Nurse staffing ratios, skill mix, and adherence to evidence-based prevention protocols have been described in the literature as factors potentially associated with pressure ulcer incidence and nursing-sensitive outcomes [7,17,18]. However, these variables were not directly measurable within the SDO administrative database.

Despite this heterogeneity, national data suggested a partial reduction in the magnitude of regional variability over time, although these patterns should be interpreted cautiously given the potential influence of coding practices and residual confounding.

The forthcoming National Guideline System (Sistema Nazionale Linee Guida, SNLG) on pressure ulcer prevention and treatment—implemented in 2025—may contribute to improving the harmonization of clinical documentation, monitoring approaches, and reporting consistency across regions [19].

### 4.3. Clinical and Organizational Characteristics (Age, Discipline, and Case-Mix)

Descriptive analyses of hospital stays provided further insight into the clinical and contextual characteristics associated with pressure ulcers. An inverse relationship was observed between patient age and length of stay (LOS): younger patients (18–39 years) experienced longer hospitalizations (mean 42.4 days) compared with older adults (≥85 years, mean 18.8 days). This pattern suggests that younger patients may represent a highly selected subgroup with complex chronic or post-traumatic conditions requiring multidisciplinary or rehabilitative care [7,10].

In contrast, shorter LOS among older patients may reflect acute management with early transitions to post-acute or long-term care settings.

Importantly, the post-2021 decline in LOS did not coincide with an increase in cases where pressure ulcers were the only secondary diagnosis, potentially reflecting changes in hospitalization patterns, discharge organization, patient case-mix, or administrative coding dynamics during the post-pandemic period.

Most hospitalizations occurred in internal medicine and rehabilitation wards—particularly General Medicine, Geriatrics, and Rehabilitation—which together accounted for nearly 80% of all cases. These units typically manage frail, chronically ill, and immobile patients at high risk of pressure injury [7,10]. While LOS remained moderate in General Medicine and Geriatrics (16–19 days), Rehabilitation and Long-Term Care units showed prolonged stays (≥35 days), reaching 104.8 days in Neurorehabilitation.

Although longer hospitalizations may increase exposure risk, these units may also differ in patient clinical complexity, duration of hospitalization, documentation practices, and organizational management approaches related to long-term care and rehabilitation pathways. Previous studies have described the implementation of structured prevention protocols and continuous wound surveillance practices in these settings, although the present study did not directly evaluate their implementation or effectiveness [2,20].

Intensive and Semi-Intensive Care Units demonstrated extended LOS, potentially consistent with higher patient acuity, immobility, and complexity of care.

In contrast, surgical specialties reported shorter stays (13–21 days), consistent with the previous literature describing enhanced recovery and “fast-track” perioperative models, which have previously been associated with reduced immobility-related complications [21].

### 4.4. Implications for Practice and Policy

The concurrent variation in hospital LOS and coded pressure ulcer rates observed during the study period may reflect multiple interacting factors, including changes in hospitalization patterns, organizational dynamics, patient complexity, discharge management, and administrative coding practices.

Several healthcare systems have increasingly adopted systemic approaches integrating risk assessment, staff education, and quality monitoring strategies [4,22], although the present study was not designed to evaluate the effectiveness of specific preventive interventions.

These findings may support the importance of further methodological development and validation of nursing-sensitive indicators, such as the adapted PSI 03-based administrative indicator, within national systems for descriptive monitoring of healthcare activity and patient safety outcomes [2,23]. However, persistent regional variability may warrant further investigation of organizational, epidemiological, and coding-related differences across healthcare systems.

The 2025 SNLG guidelines may represent an opportunity to improve the standardization of preventive approaches, clinical documentation, and monitoring practices nationwide [19].

### 4.5. Strengths and Limitations

The principal strength of this study lies in the use of a comprehensive national database encompassing more than 55 million national hospital discharge records, with approximately 10.7 million eligible hospitalizations included in the final analytic denominator, analyzed using an adapted AHRQ PSI 03-based algorithm and standardized risk-adjustment methods. The integration of descriptive, standardized, and exploratory analytical approaches provided a multidimensional overview of temporal and regional variability across the national dataset. Nonetheless, several limitations must be acknowledged. The administrative nature of SDO data limits clinical granularity, preventing ulcer staging and causal inference.

While the use of the AHRQ PSI 03-based algorithm relies on administrative discharge data (SDO), which may be affected by under-coding compared with active clinical surveillance systems, the Italian DRG-based reimbursement framework may partially mitigate this limitation. In Italy, secondary diagnoses such as Hospital-Acquired Pressure Ulcers (HAPUs) may contribute to the classification of complications or comorbidities and potentially influence DRG weighting and hospital reimbursement. Consequently, hospital administrative departments and clinical coders may have an operational incentive to accurately document these events within discharge records. Although minor pressure ulcer stages may still be under-represented, the relative consistency of the coding framework during the 2018–2024 study period may partially support the interpretability and longitudinal comparability of the observed national trends.

In addition, the SDO database does not include several clinically relevant variables commonly associated with pressure ulcer risk, including functional status, mobility impairment, nutritional status, Braden scale domains, and standardized nursing assessment variables.

Differences in coding completeness—particularly regarding the “present on admission” flag—may have influenced interregional variability. Moreover, Italy’s continued reliance on ICD-9-CM restricts comparability with countries employing ICD-10 or SNOMED systems.

### 4.6. Future Directions

Future research should focus on integrating administrative and clinical datasets to complement PSI data with detailed nursing information, including ulcer staging, preventive interventions, and care processes. Such integration may support the development of hybrid indicators capable of improving the interpretability and contextualization of nursing-sensitive outcomes within administrative healthcare databases.

Future validation studies comparing administrative indicators with clinical documentation and electronic nursing records may further improve the interpretability and validity of nursing-sensitive outcome measures.

Ongoing national studies in rehabilitation and pediatric contexts may offer valuable frameworks for implementing multidimensional outcome measurement.

The establishment of multidisciplinary wound care teams and digital monitoring systems may also improve documentation consistency and support earlier identification of pressure ulcer events.

Comparative analyses, including other nursing-sensitive outcomes such as falls and catheter-associated infections, could contribute to a broader descriptive understanding of patient safety monitoring patterns across regions.

## 5. Conclusions

In conclusion, this nationwide study represents the first comprehensive analysis of Hospital-Acquired Pressure Ulcers (HAPUs) in Italy based on the complete National Hospital Discharge Record (SDO) database from 2018 to 2024 and demonstrates the feasibility of adapting the AHRQ PSI 03 algorithm to the Italian administrative healthcare context in order to establish a standardized national baseline for monitoring nursing-sensitive outcomes.

The analyzed data revealed substantial temporal and interregional variability across the country. While these nationwide trends provide potentially valuable descriptive insights for healthcare governance and epidemiological surveillance, the observational and administrative nature of the database requires cautious interpretation. The observed variations should therefore be interpreted as exploratory epidemiological findings and speculative hypotheses potentially associated with differences in healthcare organization, preventive practices, clinical complexity, continuity of care, administrative coding behavior, and regional reporting dynamics, including those related to the Italian DRG-based reimbursement framework, rather than as direct evidence of healthcare quality performance or causal clinical effects.

Methodologically, the study supports the feasibility of using standardized national administrative databases (SDOs) for the macro-level monitoring of nursing-sensitive indicators and longitudinal epidemiological trends within the Italian healthcare system. At the same time, the findings also highlight the intrinsic limitations of administrative healthcare data, including residual confounding, variability in coding practices, and limited clinical granularity.

Future studies integrating administrative databases with clinical documentation, electronic nursing records, longitudinal linkage systems, and multicenter validation approaches will be essential to improve risk adjustment, strengthen interpretability, and better define the applicability of these indicators within the Italian healthcare performance evaluation framework. Ultimately, these findings may provide a preliminary methodological and epidemiological foundation for future targeted clinical audits, quality improvement strategies, and integrated national surveillance systems focused on hospital-acquired pressure ulcers and nursing-sensitive outcomes.

## Figures and Tables

**Table 1:** Summary of analytical components and methodological specifications.

Component	Description
Data source	National Hospital Discharge Records (SDO)
Study design	Retrospective nationwide observational study
Indicator	Adapted PSI 03-based pressure ulcer rate
ICD-9-CM codes	Secondary diagnosis codes 707.0x (70700–70709)
Eligible population	Adult ordinary inpatient admissions aged ≥ 18 years with LOS ≥ 5 days
Main exclusions	POA pressure ulcers, MDC 09 and MDC 14, selected DRGs, transfers/long-term care admissions, selected neurological/paralytic conditions, selected operating room procedures, transfer exclusions and POA-based exclusions according to AHRQ PSI 03 logic
Cohort selection flow	Sequential inclusion/exclusion process with ICD-9-CM, DRG, MDC, POA, transfer and procedural exclusions
POA management	Pressure ulcers coded as present on admission, excluded from numerator according to adapted PSI 03 specifications
Repeated admissions	Analysed as independent hospitalization episodes due to anonymised database structure
Risk model	Logistic regression, using a generalized linear model with binomial distribution and logit link (SAS 9.4)
Covariate	Age (continuous) as primary adjustment covariate
Calibration	Observed/Expected ratio (k coefficient)
Temporal analysis	Exploratory non-linear analysis using PROC NLIN in SAS with spline-based interpolation
Outcome metrics	Crude and age-adjusted rates per 10,000 eligible discharges
Confidence intervals	95% CIs derived from model standard errors

**Table 2:** National results of hospital-acquired pressure ulcers based on the ADAPTED AHRQ PSI 03 administrative indicator, 2018–2024.

Year	PSI (*n*)	Hospitalizations (*n*)	Crude Rate	Adjusted Rate	Lower CI	Upper CI
2018	3657	1,731,223	21.1	21.3	18.9	23.9
2019	2945	1,706,861	17.2	17.2	15.3	19.3
2020	1888	1,419,821	13.3	13.5	11.9	15.2
2021	1912	1,446,384	13.2	13.6	12.1	15.3
2022	2020	1,452,913	13.9	13.7	12.1	15.4
2023	2189	1,496,787	14.6	14.4	12.8	16.2
2024	2728	1,501,514	18.2	17.9	15.9	20.1

**Table 3:** Regional variability of hospital-acquired pressure ulcer rates based on the adapted AHRQ PSI 03 administrative indicator, Italy, 2018–2024.

Region	Total PSI Events (2018–2024)	Total Eligible Discharges (2018–2024)	Overall Crude Rate per 10,000 Discharges	Median Annual Age-Adjusted Rate	Range of Annual Age-Adjusted Rates
Abruzzo	1168	241,062	48.5	42.7	33.6–75.3
Apulia	930	684,405	13.6	12.2	9.6–37.0
Autonomous Province of Bolzano	123	104,569	11.8	11.4	9.3–17.0
Autonomous Province of Trento	29	107,537	2.7	2.4	0.7–6.1
Basilicata	27	105,832	2.6	2.9	0.8–4.3
Calabria	608	282,387	21.5	23.7	22.3–31.5
Campania	1345	719,671	18.7	22.7	18.9–28.8
Emilia-Romagna	634	1,024,895	6.2	4.8	3.9–12.5
Friuli Venezia Giulia	26	241,516	1.1	1.2	0.2–1.5
Lazio	5169	1,037,346	49.8	54.3	26.3–94.0
Liguria	409	365,932	11.2	10.9	7.1–14.4
Lombardy	2309	1,815,170	12.7	13.4	11.6–15.7
Marche	157	280,372	5.6	5.3	4.2–7.8
Molise	185	54,699	33.8	32.2	0.0–83.6
Piedmont	1323	721,713	18.3	21.2	8.5–35.8
Sardinia	514	279,829	18.4	20.0	13.9–27.5
Sicily	1146	781,370	14.7	19.7	12.5–23.9
Tuscany	305	694,495	4.4	3.9	2.7–6.8
Umbria	185	192,560	9.6	9.1	5.2–16.9
Aosta Valley	39	31,136	12.5	14.2	4.5–24.0
Veneto	708	993,007	7.1	7.8	3.9–9.8

Source: Authors’ elaboration based on the Italian National Hospital Discharge Records (SDO), Italy, 2018–2024 [12]. Notes: Overall crude rates were calculated using cumulative PSI events and eligible discharges across the study period. Median annual adjusted rates and annual adjusted rate ranges were derived from yearly region-specific age-adjusted estimates observed between 2018 and 2024. Each region contributed up to seven annual observations.

**Table 4:** Mean length of stay by age group (2018–2024).

Age Group (Years)	Hospitalizations (*n*)	Mean LOS (Days)
18–39	76	42.4
40–45	79	36.7
46–50	156	35.3
51–55	274	31.3
56–60	332	30.6
61–65	513	29.6
66–70	869	29.6
71–75	1529	25.9
76–80	2321	23.1
81–85	2878	20.5
≥85	8297	18.8

Source: Authors’ elaboration based on the Italian National Hospital Discharge Records (SDO), 2018–2024 [12].

**Table 5:** Mean length of stay by clinical discipline (2018–2024).

Discipline	Hospitalizations (*n*)	Mean LOS (Days)
General Medicine	9722	18.9
Geriatrics	2752	16.1
Physical Medicine and Rehabilitation	1307	36.8
Long-Term Care	657	46.5
Neurology	460	29.6
Observation Unit	390	12.6
Infectious and Tropical Diseases	399	26.3
Pulmonology	228	31.8
Intensive Care Unit (ICU)	150	38.5
Nephrology	162	23.9
General Surgery	98	20.7
Orthopedics and Traumatology	82	20.4
Oncology	75	17.9
Cardiology	67	23.9
Gastroenterology	63	30.7
Intermediate Care Unit	63	22.3
Psychiatry	43	42.0
Rheumatology	34	26.7
Hematology	28	30.3
Neurorehabilitation	21	104.8
Urology	16	13.5

Source: Authors’ elaboration based on the Italian National Hospital Discharge Records (SDO), 2018–2024 [12].

## Data Availability

The data that support the findings of this study are owned by the Italian Ministry of Health and are not publicly available due to privacy and data protection regulations. Aggregated or anonymised data may be available from the corresponding author upon reasonable request and subject to institutional approval.

## Supplementary Materials

The following supporting information can be downloaded at: https://www.mdpi.com/article/10.3390/healthcare14111492/s1, Table S1: Sequential cohort selection process and exclusion criteria applied to the adapted PSI 03 algorithm, Italy, 2018–2024.
