# Supplementary material for: Nationwide Trends in Hospital-Acquired Pressure Ulcers, 2018–2024
Source: Healthcare (Basel). 2026 May 27;14(11):1492. doi: 10.3390/healthcare14111492 (PMC13257155; doi:10.3390/healthcare14111492)
Supplement: Supplementary file 1 [file healthcare-14-01492-s001.zip › healthcare-4298367-supplementary.pdf]

Supplementary Table S1. Sequential cohort selection process and exclusion criteria applied to the adapted PSI 03 algorithm, Italy, 2018–2024

This supplementary table reports the sequential cohort selection process and exclusion criteria applied to the adapted AHRQ PSI 03-based administrative indicator using the Italian Hospital Discharge Records (SDO) database for the period 2018–2024**.**

**Step**	**Criterion applied**	**Coding/specification**	**Excluded hospitalizations (n)**	**Remaining eligible hospitalizations (n)**
1	Initial dataset	All Italian SDO records, 2018–2024	—	55,021,365
2	Exclusion of non-ordinary admissions	reg_ric ≠ 1	11,436,702	43,584,663
3	Exclusion of age <18 years	eta <18	5,497,707	38,086,956
4	Exclusion of short stays	Length of stay <5 days	17,666,611	20,420,345
5	Exclusion of principal diagnosis of pressure ulcer	ICD-9-CM 7070x in principal diagnosis	13,509	20,406,836
6	Exclusion of selected DRGs	DRGs 044, 385–391, 469–470	12,876	20,393,960
7	Exclusion of transfers and long-term care admissions	SDO provenance codes 05, 06, 07, 08, 10	2,393,880	18,000,080
8	Exclusion of neurological/paralytic conditions	ICD-9-CM paralytic and neurological conditions according to adapted PSI 03 specifications	189,219	17,810,861
9	Exclusion of selected debridement/skin graft procedures	ICD-9-CM procedures 8345, 8622, 8628, 8670–8675	127,943	17,682,918
10	Exclusion of selected operating room procedures	Adapted AHRQ PSI 03 operating room procedure exclusions	6,396,107	11,286,811
11	Exclusion of pressure ulcers present on admission (POA)	Secondary diagnosis 7070x with POA flag = 1	66,724	11,220,087
12	Exclusion of MDC 09 and MDC 14	MDC 09 (skin disorders) and MDC 14 (pregnancy/childbirth)	464,584	10,755,503
13	Final analytic denominator	Eligible hospitalizations included in PSI analysis	—	10,755,503
14	Final PSI events / numerator	Eligible hospitalizations with qualifying secondary diagnosis 707.0x	—	17,339

Abbreviations: SDO = Hospital Discharge Records; POA = Present on Admission; DRG = Diagnosis-Related Group; MDC = Major Diagnostic Category.

The adapted PSI 03 algorithm was developed according to the AHRQ PSI 03 conceptual framework and adapted to the structural characteristics of the Italian ICD-9-CM 2007 administrative coding system. Because ulcer staging codes were not consistently available within the SDO database, the indicator should be interpreted as an adapted PSI 03-based administrative indicator rather than a direct implementation of the original AHRQ PSI 03 specification.
